# TSG-6 promotes healing of critical-sized bone defects in mice

**DOI:** 10.3389/fimmu.2025.1712152

**Published:** 2025-11-28

**Authors:** Oliver Küppers, Mubashir Ahmad, Sandra Dieterich, Jasmin Maria Bülow, Christoph Kölbl, Melanie Haffner-Luntzer, Karin Scharffetter-Kochanek, Anita Ignatius, Verena Fischer

**Affiliations:** 1 Institute of Orthopaedic Research and Biomechanics, Ulm University Medical Center, Ulm, Germany; 2 Department of Dermatology and Allergic Diseases, Ulm University, Ulm, Germany

**Keywords:** TSG-6, bone healing, mesenchymal stem cells, osteogenic differentiation, M2 macrophage polarization, inflammation

## Abstract

Tumor necrosis factor-stimulated gene 6 (TSG-6) is recognized for its anti-inflammatory and pro-regenerative properties in various inflammatory conditions, including peritonitis, arthritis, traumatic brain injury, and corneal as well as skin wound healing. However, its regenerative role in bone metabolism and repair remains largely unexplored. This study aimed to address this gap. We observed an increased expression of *Tnfaip6* (*Tsg-6*) during early osteogenic differentiation of murine mesenchymal stem cells (mMSCs). Silencing *Tnfaip6* significantly impaired osteogenic differentiation, as indicated by reduced alkaline phosphatase activity and downregulation of key osteogenic marker genes. RNA-seq analysis of *Tnfaip6*-deficient mMSCs revealed decreased expression of several genes that are critical for bone formation and healing, including matrix metallopeptidase 13, decorin, membrane metallopeptidase, and integrin binding sialoprotein. Conversely, gremlin 1, a known inhibitor of osteoblastogenesis, was significantly upregulated. To investigate the *in vivo* relevance, we assessed the effects of locally delivered recombinant TSG-6 (rTSG-6), embedded in a collagen gel, on bone regeneration in a 1.5 mm critical-sized femoral defect model in male C57BL/6J mice. Treatment with 50 μg rTSG-6 significantly enhanced bone formation compared to untreated and low-dose (10 µg) controls at 35 days post-injury. This effect was associated with increased osteoblast activity and reduced osteoclast activity. Moreover, rTSG-6 promoted M2 macrophage polarization and increased T-helper cell infiltration at the defect site three days after injury. In summary, TSG-6 enhances bone regeneration in critical-sized bone defects, through immunomodulation, particularly via M2 macrophage polarization, and stimulation of osteogenesis. These findings identify TSG-6 as a promising therapeutic candidate to improve bone repair, for example, in patients with osteoporosis, concomitant trauma or large bone defects.

## 1 Introduction

Bone healing is a complex, tightly regulated process comprising overlapping phases of inflammation, repair, and remodeling, during which cellular and molecular mechanisms restore bone integrity and allow the full recovery of strength, mobility, and function (1, 2). While most bone fractures heal uneventfully with appropriate treatment, challenges arise in patients with comorbidities such as chronic bone disorders (e.g., osteoporosis), aging, concomitant trauma, or large bone defects, leading to delayed healing or non-unions in 5-10% of cases (3, 4). Current treatments for impaired fracture healing primarily involve surgical interventions, while pharmacological options remain limited (5, 6). To date, only one clinically approved osteoanabolic growth factor, bone morphogenetic protein-2 (BMP-2), is in clinical practice (7–9). Additional strategies include transplantation of mesenchymal stem cells (MSCs) or the use of biomaterials, often applied in combination (10, 11). However, these approaches are limited by modest efficacy, adverse effects, and high costs (5, 12). Therefore, the development of novel therapeutic approaches remains a major clinical need.

Tumor necrosis factor-stimulated gene-6 (TSG-6), encoded by *TNFAIP6*, has attracted considerable attention for its anti-inflammatory and pro-regenerative properties (13). It is a multifunctional protein secreted by various cell types, including monocytes, neutrophils, MSCs, chondrocytes, and fibroblasts, in response to inflammatory stimuli (e.g., tumor necrosis factor α, interleukin-1β (IL-1β), lipopolysaccharide, hypoxia, oxidative stress), growth factors (e.g., transforming growth factor β (TGF-β), fibroblast growth factor), or mechanical injury. TSG-6 is highly conserved across species, with 94% sequence identity between human and murine orthologs (13). Functionally, it exerts both anti-inflammatory and regenerative effects, improving outcomes in multiple inflammatory disease models, including chronic liver disease (14), peritonitis (15), traumatic brain injury (16), corneal injury (17), osteoarthritis (18), and wound healing (19). These effects are mediated by diverse mechanisms, such as attenuating inflammation by enhancing autophagy (14), suppressing pro-inflammatory mediators (17, 20, 21), inhibiting neutrophil infiltration (16, 22), and promoting a shift in macrophage polarization from pro-inflammatory M1 to the anti-inflammatory M2 phenotype (15, 21, 23). Moreover, TSG-6 can bind and modulate extracellular matrix components, thereby regulating chemokine signaling and immune cell recruitment (24). MSCs represent a particularly relevant source of TSG-6, mediating many of its regenerative effects. For example, in a murine model of full-thickness skin wounds, MSC-derived TSG-6 accelerated wound closure and reduced fibrosis by dampening macrophage activation (19). Notably, wound and bone healing share common phases of inflammation, repair, and remodeling. A tightly regulated inflammatory response is also essential for successful bone healing, while excessive or prolonged inflammation, particularly sustained neutrophil and M1 macrophage activity, impairs healing (1, 25). Based on these parallels, we hypothesize that TSG-6 also contributes to bone regeneration; however, to the best of our knowledge, its role in bone repair has not been previously investigated.

Only a few studies have investigated the role of TSG-6 in bone biology. TSG-6 knockout (*Tnfaip6^-/-^)* mice displayed an increased bone mass due to reduced osteoclast activity (26). Furthermore, MSCs isolated from *Tnfaip6^-/-^* mice failed to differentiate into adipocytes and osteoblasts, while treatment with recombinant TSG-6 restored adipogenic but not osteogenic differentiation (27). In addition, adenoviral overexpression of TSG-6 induced ectopic bone formation in mice (28). Collectively, these findings suggest that TSG-6 regulates bone homeostasis and may also influence bone healing.

Therefore, in this study, we examined the role of TSG-6 in osteogenic differentiation *in vitro* and explored associated underlying molecular mechanisms. Furthermore, we investigated whether local administration of TSG-6 enhances the healing of critical-sized bone defects in mice, with particular emphasis on its immunomodulatory and pro-regenerative effects. Our results highlight TSG-6 as a promising therapeutic candidate for enhancing bone regeneration.

## 2 Materials and methods

### 2.1 *In vitro* experiments

#### 2.1.1 Cell culture

To investigate the potential role of *Tsg-6* in the osteogenic differentiation of murine mesenchymal stem cells (mMSCs), we isolated primary mMSCs from the long bones of 12-week-old male C57BL/6J mice (o.135-7; Regierungspräsidium Tuebingen, Germany), as previously described (29). The cells were resuspended in a murine proliferation medium consisting of DMEM-F12 (Thermo Fisher Scientific; Waltham, MA, USA; Cat. 11320033) supplemented with 15% fetal bovine serum (FBS) superior (Merck, Darmstadt, Germany; Cat. S0615), 1% L-glutamine (Thermo Fisher Scientific; Cat. 25030-024), 50 μM β-mercaptoethanol (Thermo Fisher Scientific; Cat. 21985023), 1% penicillin/streptomycin, and 0.5% amphotericin B (Sigma-Aldrich, St. Louis, MO, USA; Cat. A2942). The cultured cells reached 60%–70% confluency within two weeks. Then, osteogenic differentiation was induced with osteogenic medium containing DMEM-F12 (Thermo Fisher Scientific), 15% FBS superior (Merck; Cat. S0615), 1% penicillin/streptomycin, 100 μg/mL (+)-sodium L-ascorbate (Sigma-Aldrich, USA), and 5 mM β-glycerophosphate (Sigma-Aldrich).

#### 2.1.2 Cell viability assay, alkaline phosphatase and alizarin red S staining

Cell viability was measured using the PrestoBlue cell viability reagent (Thermo Fisher Scientific; Cat. A13261) according to the manufacturer’s instructions after 3, 6 and 12 days of osteogenic induction. Following the cell viability assay and washing with PBS, quantitative alkaline phosphatase (ALP) staining was conducted using the Amplite colorimetric ALP assay kit *yellow color* (Biomol, Hamburg, Germany; Cat. ABD-11950) as recommended by the manufacturer. For qualitative ALP staining on day 6 of osteogenic induction, cells were fixed with citrate-acetone-formaldehyde (Sigma-Aldrich) and incubated with the ALPL staining solution (Sigma-Aldrich) for 30 min in the dark at RT, followed by imaging. Quantitative Alizarin Red S (ARS) staining was performed on day 27 of osteogenic differentiation using 0.25 mL of 1% w/v ARS solution (Sigma-Aldrich) as described previously (30). For quantification, cells were incubated with 10% acetic acid on a plate shaker (dark, RT, 30 min), followed by monolayer harvest, heating, and cooling. The supernatants were then collected and neutralized with 10% ammonium hydroxide, and the optical density (OD) was measured at 405 nm using a Tecan plate reader (Tecan, Männedorf, Switzerland).

#### 2.1.3 Small interfering RNA transfection experiments

*Tsg-6* specific knockdown experiments were carried out using non-targeting control siRNA (Silencer Negative Control No.1 siRNA, Thermo Fisher Scientific; Cat. 4404021) and siRNA targeting *Tnfaip6* (*Tsg-6*) (Silencer Select siRNA, Thermo Fisher Scientific; Cat. 4390771). si*Tnfaip6* or non-targeting siRNA (siNT) was incubated with 1× siRNA buffer, Lipofectamine RNAiMAX transfection reagent (Thermo Fisher Scientific; Cat. 13778 075), and Opti-MEM (Life Technologies, Carlsbad, CA, USA; Cat. 31985 047) for 15 min at room temperature. mMSCs were resuspended in transfection medium containing DMEM-F12 (Thermo Fisher Scientific), supplemented with 15% FBS superior (Merck). Reverse transfection was performed using 0.125% Lipofectamine RNAiMAX and a final siRNA concentration of 20 nM for 48 h.

#### 2.1.4 RNA isolation, complementary DNA synthesis, and quantitative reverse transcription-polymerase chain reaction

Total RNA was isolated from cells using the RNeasy™ kit (Qiagen, Hilden, Germany; Cat. 74104) according to the manufacturer’s instructions. cDNA synthesis was performed with the Omniscript reverse transcriptase kit (Qiagen; Cat. 205113). Quantitative real-time PCR (qRT-PCR) was conducted using the QuantStudio 3 system (Applied Biosystems, Waltham, MA, USA). Gene expression levels of *Tnfaip6* and osteogenic marker genes (runt-related transcription factor 2 (*Runx2*), Sp7 transcription factor 7 (*Sp7*), alkaline phosphatase (*Alpl*), and bone gamma-carboxyglutamate protein (*Bglap*)) were analyzed using the delta-delta CT method and normalized to the housekeeping gene ß-actin *(Actb)*. A list of murine primer sequences used in this study is provided in **Supplementary Table 1**.

#### 2.1.5 RNA-seq and bioinformatics analysis

RNA samples with an RNA integrity number greater than 9.5 were selected for RNA-seq analysis, which was carried out by Novogene (Novogene Company Limited, Cambridge, UK). Briefly, sequencing reads were mapped to the murine reference genome (ensembl_mus_musculus_grcm38_p6_gca_000001635_8), and gene quantification was performed using the fragments per kilobase of transcript per million mapped reads method. To evaluate sample repeatability and variability, a principal component analysis (PCA) plot was generated using R and RStudio with the pcaExplorer package. Differentially expressed genes (DEGs) were analyzed using RStudio and DESeq2, and the results were visualized through Enhanced Volcano. DEGs were identified based on a log2-fold change (log2FC) threshold of ±1.0 and an adjusted p-value of <0.01. Additionally, gene ontology analysis was performed using Metascape. To identify the most regulated genes within the “extracellular matrix organization,” a log fold change (M) versus mean expression (A) (MA) plot was generated to visualize DEGs. RNA-Seq datasets are provided in (**Supplementary Tables 1**–**3**) and have been deposited in the Zenodo repository (DOI: https://doi.org/10.5281/zenodo.17176977).

### 2.2 *In vivo* experiments

#### 2.2.1 Animals and study approval

Male C57BL/6J mice were purchased from Charles River Laboratories (Sulzfeld, Germany) and housed in groups of three to five mice under standard rodent conditions in the Animal Facility of Ulm University (Ulm, Germany). The mice were fed with water and a standard mouse feed *ad libitum* (Sniff R/M-H, V1535-300, Ssniff GmbH, Soest, Germany). All animal experiments complied with international regulations for the care and use of laboratory animals (ARRIVE guidelines and EU Directive 2010/63/EU for animal experiments) and were approved by the Local Ethical Committee (Nos. 1485, 1422, 1149 and 1032, Regierungspräsidium Tübingen).

#### 2.2.2 Immunohistochemistry

Tsg-6 protein was stained in 4-μm-thick longitudinal sections of paraffin-embedded fractured femora (0.44 mm gap) of 12-week-old male C57BL/6J mice up to day 21 after fracture. Antigen retrieval was performed using 0.05% trypsin, followed by overnight Tsg-6 staining (1:100; MyBioSource, San Diego, CA, USA, Cat. MBS3002899) at 4°C. For signal detection, horseradish peroxidase (HRP)-conjugated streptavidin (Vector Laboratories, Burlingame, CA, USA; Cat. PK-6100; VECTASTAIN^®^ ELITE ABC-HRP Kit) was used according to the manufacturer’s recommendations. The Vector^®^ NovaRED^®^ Substrate Kit (Vector Laboratories, Cat. Sk4800, Peroxidase (HRP)) served as chromogen, and counterstaining was performed using hematoxylin (1:2000; Waldeck, Münster, Germany; Cat. 2C-306). Species-specific non-targeting immunoglobulin was used as isotype control. The staining intensity was quantified using Fiji software.

#### 2.2.3 Study design and surgical procedure

To study effects of TSG-6 on critical-sized bone defect healing, recombinant TSG-6 protein (rTSG-6) was embedded into a collagen type-1 gel (Amedrix GmbH, Esslingen am Neckar, Germany), which was prepared according to **Supplementary Table 2** in a final gel concentration of 5 mg/mL containing either 10 µg or 50 µg rTSG-6. The gel was transplanted into the osteotomy gap of 12-week-old male C57BL/5J mice, which underwent a 1.5 mm critical-sized femoral defect. For analgesia, 25 mg/L tramadol hydrochloride (Gruenenthal GmbH, Aachen, Germany) was administered in drinking water from one day prior to surgery until three days post-surgery. Under general anesthesia with 2% isoflurane (Baxter International, Deerfield, IL, USA; Cat. HDG9623), mice received a subcutaneous. injection of clindamycin-2-dihydrogenphosphate (45 mg/kg body weight; MIP Pharma GmbH, Blieskastel, Germany; Cat. 10398274) and a subcutaneous injection of 500 μL of sodium chloride (Fresenius Kabi, Bad Homburg, Germany; Cat. 1312811) prior to surgery. The femoral critical-sized defect (1.5 mm) was created as previously described (31). Briefly, after lateral exposure of the femur, an external fixator (linear axial stiffness: 18.1 N/mm, torsional stiffness: 1.5 N/mm; RISystem, Davos, Switzerland) was mounted and a 1.5 mm defect was created using a saw jig and 0.22 mm Gigli wire saws (RISystem). Mice were randomly assigned to one of the following groups: empty defect, PBS (vehicle)-loaded collagen gel, or rTSG-6-loaded collagen gel. Mice were euthanized at different time points post-surgery via isoflurane overdose following cardiac blood withdrawal, and all analyses were performed in a blinded manner. Exclusion criteria included unphysiological limb loading or weight loss; however, no animals met these criteria.

#### 2.2.4 Human TSG-6 enzyme-linked immunosorbent assay

To assess the release kinetics of rTSG-6 from the gel, collagen gels loaded with 1 μL of rTSG-6 [1 mg/mL] were incubated in PBS and stored at 37 °C under constant agitation. The supernatant was collected at defined time points (30 min, 1 h, 2 h, 4 h, 8 h, 1 d, 2 d, 4 d, 10 d, 35 d) and rTSG-6 concentrations were determined using a TSG-6 enzyme linked immunosorbent assay (ELISA) (RayBio^®^, Peachtree Corners, GA, USA; Human TSG-6 ELISA Kit; Cat. ELH-TSG6) according to the manufacturer’s instructions.

#### 2.2.5 X-ray imaging and micro-computed tomography analysis

On day 35 post-surgery, fractured femora were imaged using X-ray (Faxitron MX20; Faxitron, Tucson, AZ, USA) at 35 kV and then fixed in 4% paraformaldehyde. μCT scanning was performed at 50 kV and 200 mA using the Skyscan 1172 (Bruker, Kontich, Belgium), with an isotropic voxel resolution of 8 μm. Three-dimensional analysis was conducted according to ASBMR guidelines (32) using computed tomography analysis (CTAn) and CT volume (CTVol) software. The region of interest was defined as the newly formed bone within the initial fracture gap between the intact cortices. Two phantoms with defined hydroxyapatite (HA) contents of 250 and 750 mgHA/cm³ were used for normalization. The threshold for mineralized cortical bone tissue was set at 642 mgHA/cm^3^. Three-dimensional (3D) images were generated using CTvox software (v3.3.1). The bony bridging score was assessed using coronal and sagittal views of the 3D reconstruction images. A bridged cortex was given 1 point, while the absence of cortical bridging was scored as 0. Based on the total score, fractures were classified as healed (≥3 points) or non-healed (≤2 points).

#### 2.2.6 Histomorphometry

After μCT scanning, the fractured femora underwent decalcified histology, as previously described (33). Then, 4-μm-thick longitudinal paraffin-embedded sections were stained with toluidine blue or tartrate-resistant alkaline phosphatase (TRAP). Osteoblasts and osteoclasts were quantified within the newly formed bone in the initial fracture gap (excluding cortical bone) using Osteomeasure system (OsteoMetrics, Decatur, GA, USA) and light microscopy (Zeiss Axiophot, Carl Zeiss AG, Oberkochen, Germany). Osteoblasts were identified in toluidine blue-stained sections by their cubic-shaped morphology and attachment to the bone surface, while osteoclasts were identified by their positive TRAP staining, distinct ruffled border, and more than two nuclei.

#### 2.2.7 Fluorescence-activated cell sorting analysis

To investigate the effect of 50 µg TSG-6 on innate and adaptive immune cell populations during early fracture healing, FACS analysis was performed on the fracture hematoma, bone marrow, and spleen at days 1 and 3 post-fracture. All samples were passed through a 70-µm cell strainer (Corning Inc., Durham, NC, USA) with 10 mL of PBS yielding a single-cell suspension. Erythrolysis was performed on the bone marrow and spleen cells using erythrolysis buffer (150 nM NH_4_Cl, 1 mM KHCO_3_, 0.1 mM Na_2_EDTA) at 37°C for 5 min. Immune cell populations were stained for 30 min on ice using the antibodies listed in **Supplementary Table 3**. The following cells were identified on days 1 and 3 post-surgery: T-lymphocytes (CD3+), T-helper lymphocytes (CD3+/CD4+), cytotoxic T-lymphocytes (CD3+/CD8+), neutrophils (CD11b+/Ly6G+), and macrophages (CD11b+/F4/80+). On day 1, B-lymphocytes (CD19+) were identified, while on day 3 post-fracture, macrophages were further classified as inflammatory M1 (CD11b+/F4/80+/Arginase1+) or anti-inflammatory M2 (CD11b+/F4/80+/Arginase1-). Live-dead discrimination was performed using 7-aminoactinomycin D, and corresponding isotype controls were included (**Supplementary Table 3**). Measurements were performed on a BD FACSLyric flow cytometer (BD, Franklin Lakes, NJ, USA), and data were analyzed using FlowJo software (FlowJo LLC, Ashland, OR, USA) following a previously published gating strategy (34).

#### 2.2.8 Multiplex cytokine analysis

To investigate systemic effects of rTSG-6 treatment, serum samples were obtained on days 1 and 3 post-surgery. Inflammatory mediator concentrations were determined using a mouse Multiplex ProcartaPlex Simplex Kit (Thermo Fisher Scientific) according to the manufacturer’s guidelines. Data analysis was performed using the Bio-RadTM Bio-Plex System (Bio-Rad Laboratories, Hercules, CA, USA).

#### 2.2.9 Statistics

Results are displayed as box-and-whisker plots, showing the minimum to maximum range along with all individual data points plotted. Data in the tables are expressed as mean ± standard deviation. Statistical differences were assessed using Student’s t-test for two groups and one-way analysis of variance (ANOVA) followed by Fisher’s LSD *post hoc* test for more than two groups, performed in GraphPad Prism 9 software (GraphPad Software, Boston, MA, USA). A p-value < 0.05 was considered statistically significant. The sample calculation was guided by data from earlier studies (*in vitro*: n = 4 per group; *in vivo*: n = 8 per group).

## 3 Results

### 3.1 *Tsg-6* promotes osteoblastogenesis in murine MSCs *in vitro*

**Figure 1:**
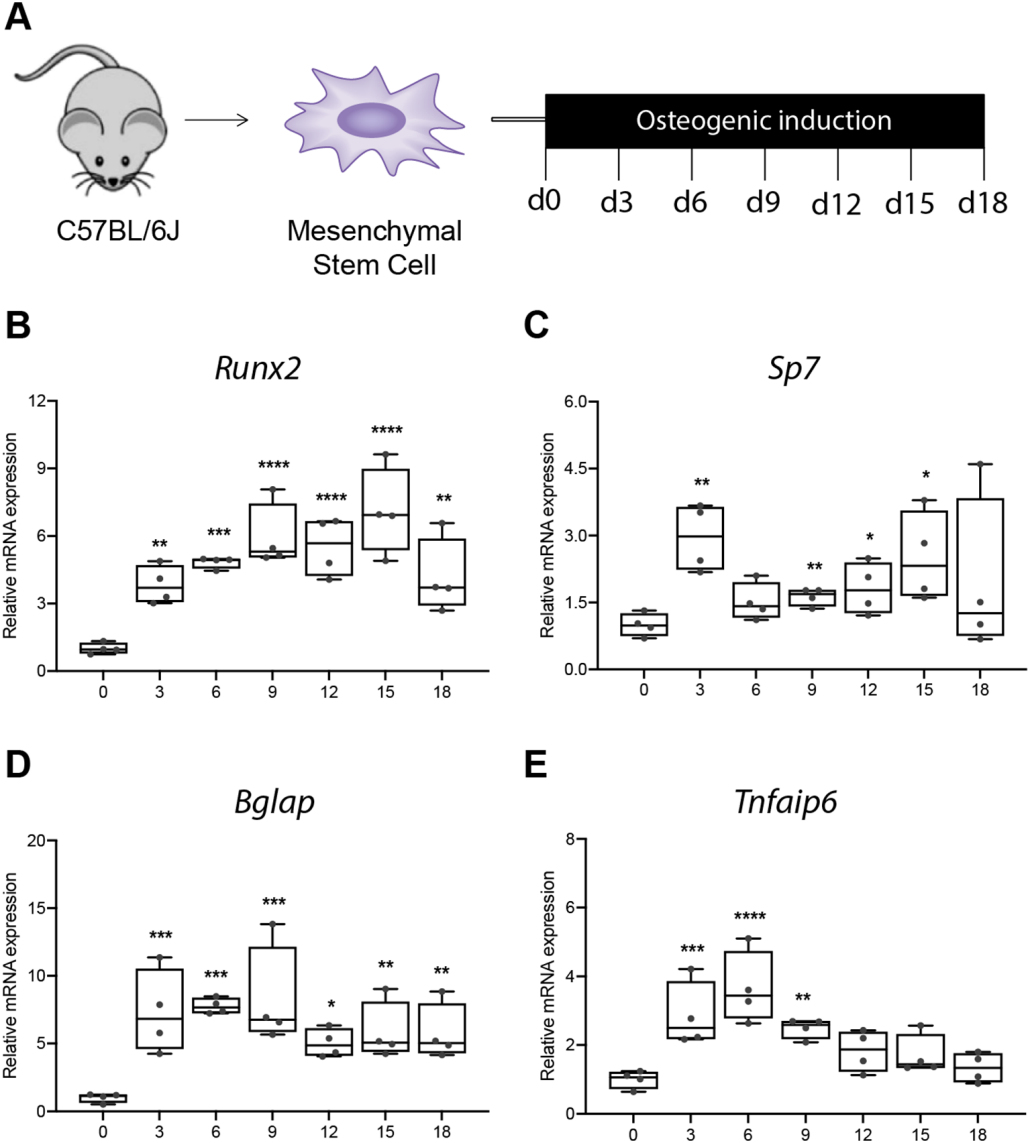
*Tsg-6* (*Tnfaip6*) gene expression during osteogenic differentiation of mMSCs. **(A)** Experimental design. Gene expression of the osteoblast-specific markers **(B)** Runt-related transcription factor 2 (*Runx2*), **(C)** SP7 transcription factor (*Sp7*), **(D)** bone gamma carboxyglutamate protein (*Bglap*) and **(E)** tumor-necrosis factor-stimulated gene 6 *(Tnfaip6)* during osteogenic differentiation of mMSCs. n = 4 per group. Statistical differences compared to day 0 were determined using ANOVA with Fisher’s LSD *post hoc* test. *p < .05, ***p < .001, ****p < .0001.

To investigate the role of *Tsg-6* in MSC osteogenic differentiation, we first analyzed its gene expression during osteoblastogenesis in mMSCs *in vitro* (**Figure 1A**). Successful osteogenic induction was confirmed by significant upregulation of osteoblast-specific marker genes, including *Runx2*, *Sp7*, *Alpl*, and *Bglap* up to day 18 (**Figures 1B-D**). Notably, *Tnfaip6* gene expression was also significantly increased from day 3 to 9, with peak levels on day 3 and 6 of osteogenic induction (**Figure 1E**). These results suggest that *Tsg-6* may play a role in the early phase of osteogenic differentiation, leading us to focus subsequent experiments on days 3 and 6.

**Figure 2:**
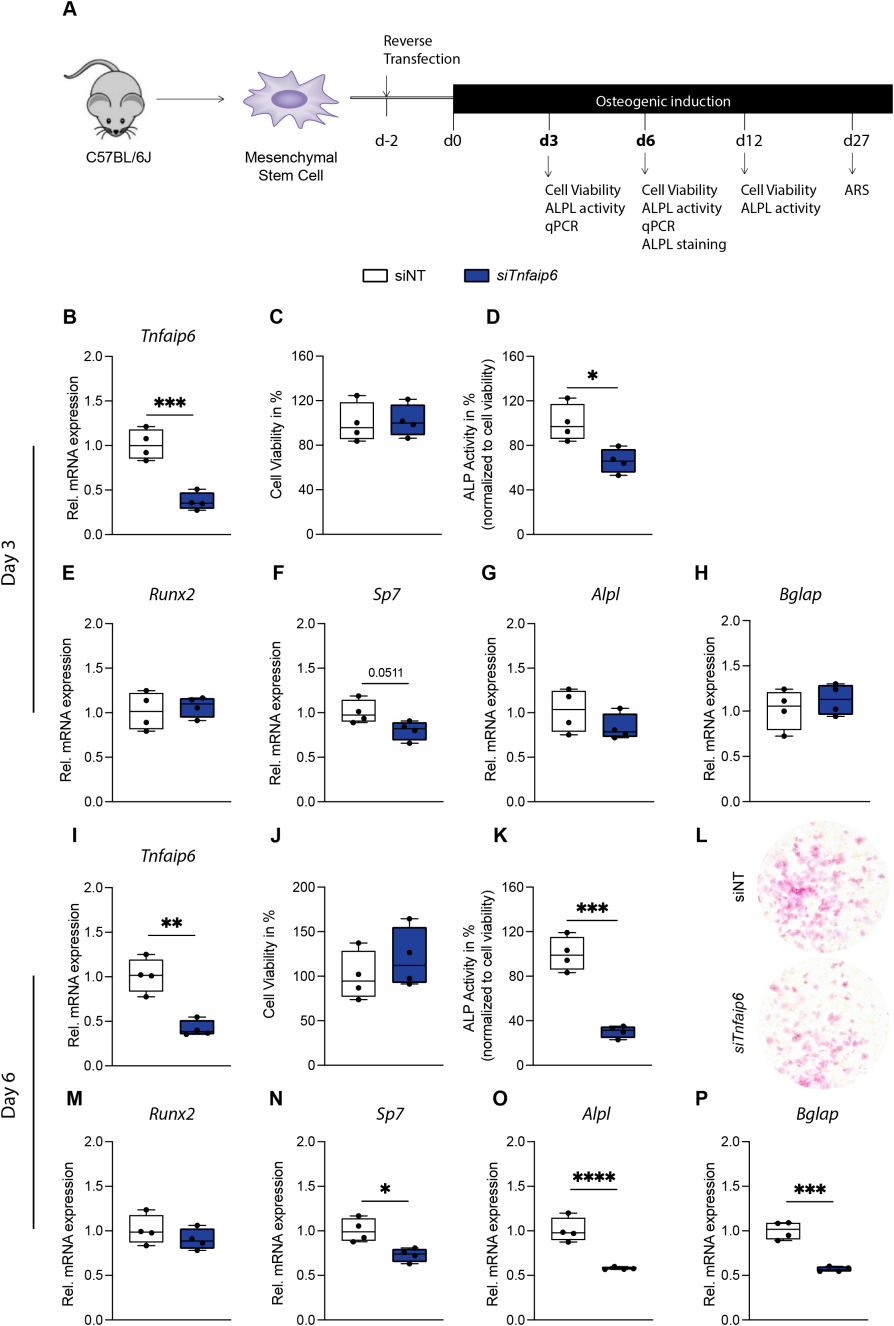
Effect of siRNA-mediated *Tsg-6* (*Tnfaip6*) knockdown on early osteogenic differentiation of mMSCs. **(A)** Experimental design. **(B)** Gene expression of *Tnfaip6*, **(C)** relative cell viability, **(D)** quantitative alkaline phosphatase (ALP) staining, and **(E–H)** gene expression of early stage (Runt-related transcription factor 2 (*Runx2*), SP7 transcription factor (*Sp7*), alkaline phosphatase (*Alpl*), and late-stage (bone gamma carboxyglutamate protein (*Bglap*)) osteoblast-specific marker genes on day 3 of osteoblast differentiation. **(I)** Gene expression of tumor-necrosis factor-stimulated gene 6 *(Tnfaip6)*, **(J)** relative cell viability, **(K)** quantitative, and **(L)** qualitative ALP staining, and **(M–P)** gene expression of early stage (*Runx2*, *Sp7*, *Alpl*) and late-stage (*Bglap)* osteoblast-specific marker genes on day 6 of osteoblast differentiation. n = 4 per group. Statistical differences between two groups were determined using unpaired Student’s *t*-test. *p < .05, **p < .01, ***p < .001, ****p < .0001.

To determine whether *Tsg-6* directly regulates osteogenic differentiation in mMSCs, we silenced *Tnfaip6* using siRNA (**Figure 2A**), achieving a transfection efficiency of 73% in *siTnfaip6* treated cells compared to siNT-treated controls (**Supplementary Figure 1**). On day 3 of osteogenic induction, knockdown efficiency remained at 63% (**Figure 2B**). While relative cell viability was unaffected (**Figure 2C**), relative ALP activity was significantly reduced in si*Tnfaip6*-treated mMSCs compared to controls (**Figure 2D**). However, the expression of osteoblast-specific marker genes, including *Runx2*, *Sp7*, *Alpl*, and *Bglap*, showed no significant changes at this time point (**Figures 2E-H**). On day 6 of osteogenic induction, the knockdown efficiency remained at 58% (**Figure 2I**), with unchanged cell viability (**Figure 2J**), but with significantly reduced relative ALP activity and ALPL staining in si*Tnfaip6*-treated mMSCs compared to controls (**Figures 2K, L**). The reduced mineralization correlated with a significant decrease in the expression of *Sp7*, *Alpl*, and *Bglap*, while *Runx2* expression remained unaffected (**Figures 2M-P**). Moreover, we observed a significantly reduced quantitative ALP activity on day 12 with unchanged cell viability (**Supplementary Figure 2A-C**), as well as a significantly decreased quantitative ARS staining on day 27 (**Supplementary Figure 2D**). In summary, these data suggest that *Tsg-6* positively regulates osteogenic differentiation in mMSCs, and its absence impairs both early and late osteoblast differentiation.

### 3.2 Identification of genes regulated by *Tsg-6* knockdown in mMSCs

**Figure 3:**
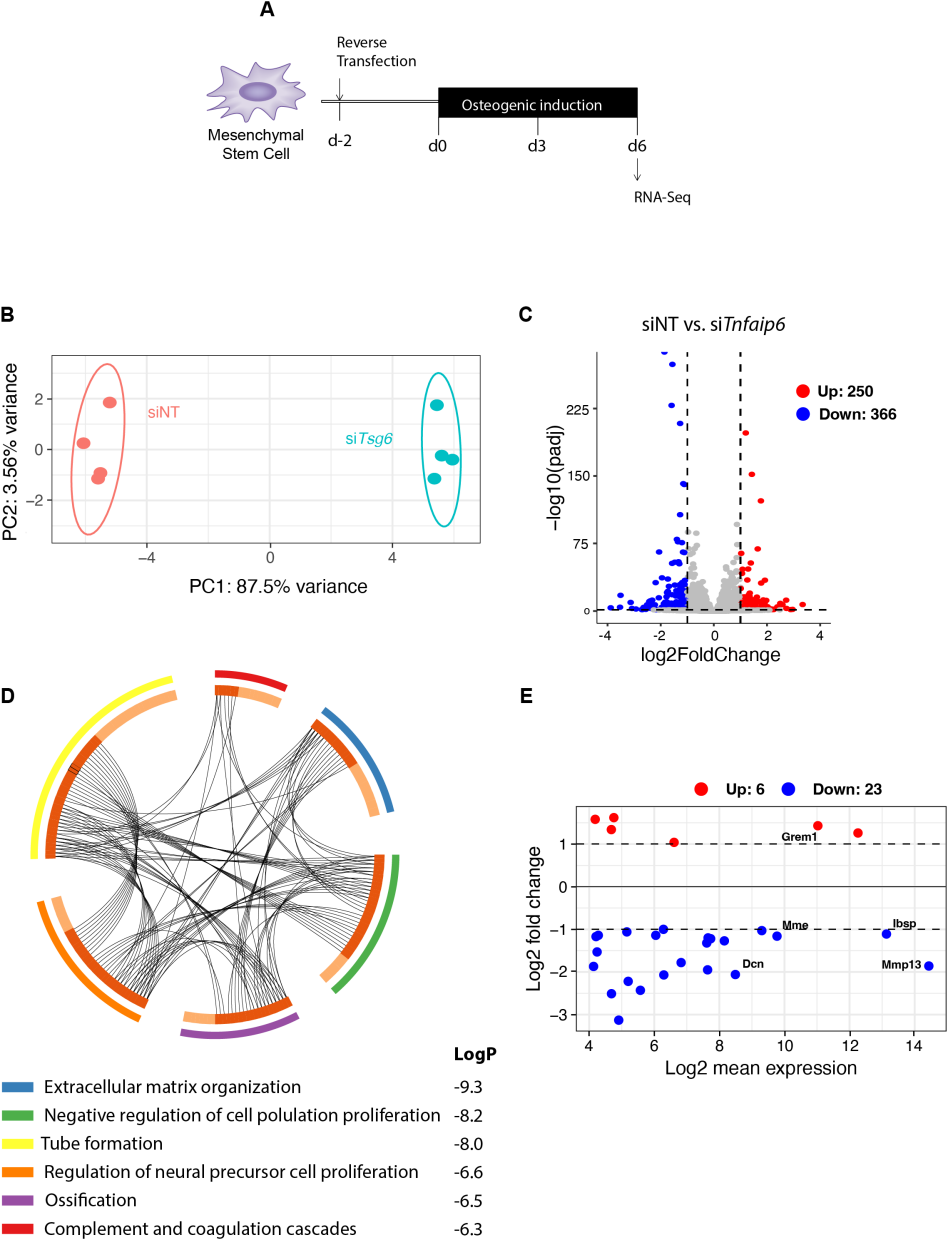
RNA-Seq analysis of *Tsg-6* (*Tnfaip6*) knockdown in mMSCs on day 6 of osteoblast differentiation. **(A)** Experimental design. **(B)** Principal component (PC) analysis and **(C)** Volcano plot of the RNA-seq data of *siTnfaip6* and siNT (control)-treated mMSCs on day 6 of osteoblast differentiation. **(D)** The top ten regulated signaling pathways identified by signaling pathway analysis of the 616 differentially regulated genes (DEGs) are shown in the circular plot. **(E)** MA-plot analysis was performed on the DEGs involved in the top-regulated pathway “extracellular matrix organization”, identifying the top 5 regulated DEGs: decorin (*Dcn*), membrane metalloendopeptidase (*Mme*), gremlin 1 (*Grem1*), integrin binding sialoprotein (*Ibsp*), and matrix metalloproteinase 13 (*Mmp13*). For statistical analysis, a threshold of log_2_ fold change (log_2_FC) ± 1.0 and an adjusted *p*-value of <.01 was applied.

To further unravel the role of *Tsg-6* in osteogenic differentiation and to identify molecular signaling pathways involved, RNA-seq analysis was performed on si*Tnfaip6*-treated mMSCs on day 6 of osteogenic induction (**Figure 3A**). The PCA plot showed clear separation between the si*Tnfaip6* and control groups, with a variance of 87.5% in PC1 and 3.56% in PC2 (**Figure 3B**). A total of 366 significantly down- and 250 upregulated DEGs were identified (**Figure 3C**). Pathway analysis using Metascape identified six highly enriched pathways including extracellular matrix organization, negative regulation of cell population, tube formation, regulation of neural precursor cell proliferation, ossification, and complement and coagulation cascades (**Figure 3D**). Given its significance, we focused on the “extracellular matrix organization” pathway (**Figure 3E**). Among the most significant DEGs, matrix metalloproteinase 13 (*Mmp13*), integrin binding sialoprotein (*Ibsp*), membrane metalloendopeptidase (*Mme*), and decorin (*Dcn*) were downregulated, while gremlin-1 (*Grem1*) was upregulated in the absence of *Tnfaip6*, all of which are known to regulate osteoblastogenesis.

### 3.3 Tsg-6 protein is expressed during bone fracture healing in mice

**Figure 4:**
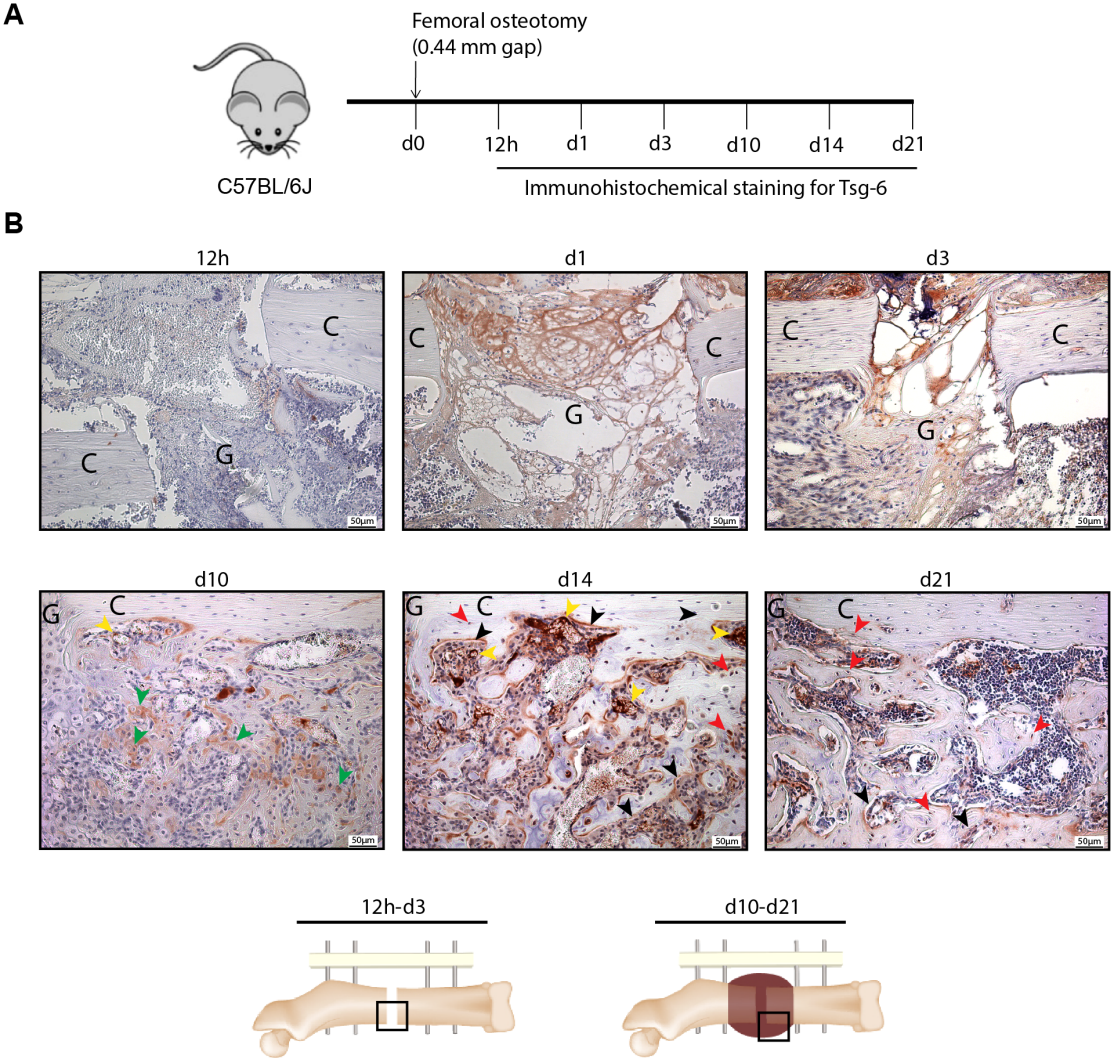
Immunohistochemical staining for Tsg-6 during fracture healing in mice. **(A)** Experimental design. **(B)** Representative images of Tsg-6 staining in the fracture gap (12 h, 1 d, 3 d) and external fracture callus (10 d, 14 d, 21d) of fractured femora, showing Tsg-6 expression in hypertrophic chondrocytes (green arrows), in osteoblasts (black arrows), osteocytes (red arrows), and vascular endothelial cells (yellow arrows). Scale bars are displayed in the images. Gap (G), cortex (C).

Based on our *in vitro* findings, we next examined Tsg-6 protein expression during regular bone fracture healing in mice (**Figure 4A**). During the early inflammatory phase of fracture healing (12 h, 1 d, and 3 d post-surgery), Tsg-6 staining was primarily observed in the granulation tissue in the fracture gap (**Figure 4B**). During the later phases of bone repair and remodeling (10 d, 14 d, and 21 d post-surgery), Tsg-6 staining was detected in hypertrophic chondrocytes, osteoblasts, and osteocytes within the fracture callus. Additionally, Tsg-6 was present in vascular endothelial cells within the callus (**Figure 4B**). Moreover, quantitative analysis suggested that Tsg-6 expression peaked during endochondral ossification (day 10) (**Supplementary Figure 3**).

### 3.4 rTSG-6 enhances critical-sized bone defect healing in mice

**Figure 5:**
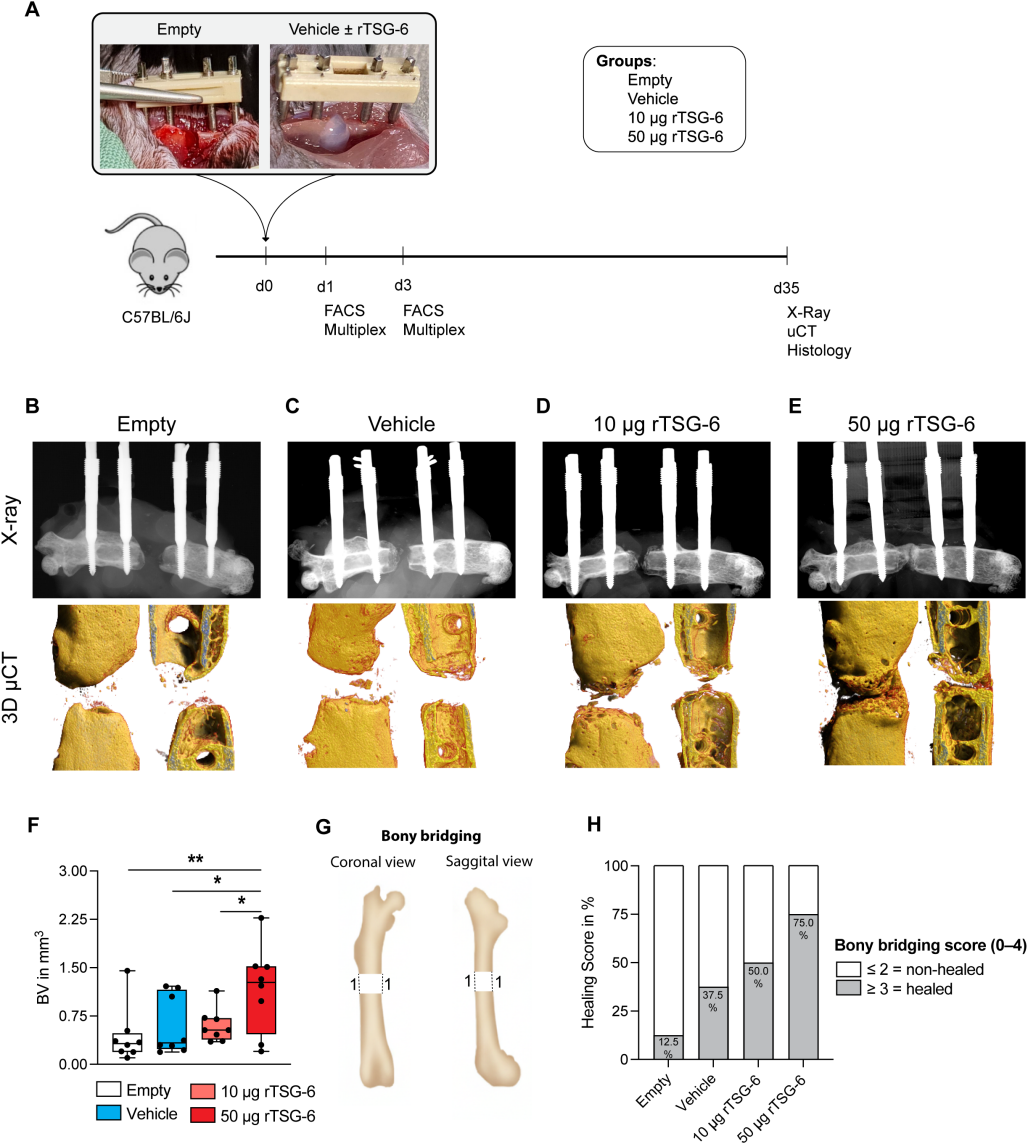
Effects of recombinant TSG-6 (rTSG-6) on critical-sized bone defect healing in mice. **(A)***In vivo* experimental design. For the pilot study, 10 µg and 50 µg rTSG-6 were loaded in a collagen type-1 gel locally delivered to the fracture site and healing was evaluated on day 35. **(B-E)** Representative X-ray images and 3D reconstruction µCT images of the four treatment groups. **(F)** Bone volume within the defect area. **(G)** Schematic illustration of the scoring criteria for bony bridging and union. **(H)** Bony bridging score. n = 8 per group. Statistical differences between the groups were determined using ANOVA with Fisher’s LSD *post hoc* test. *p < .05, **p < .01.

**Figure 6:**
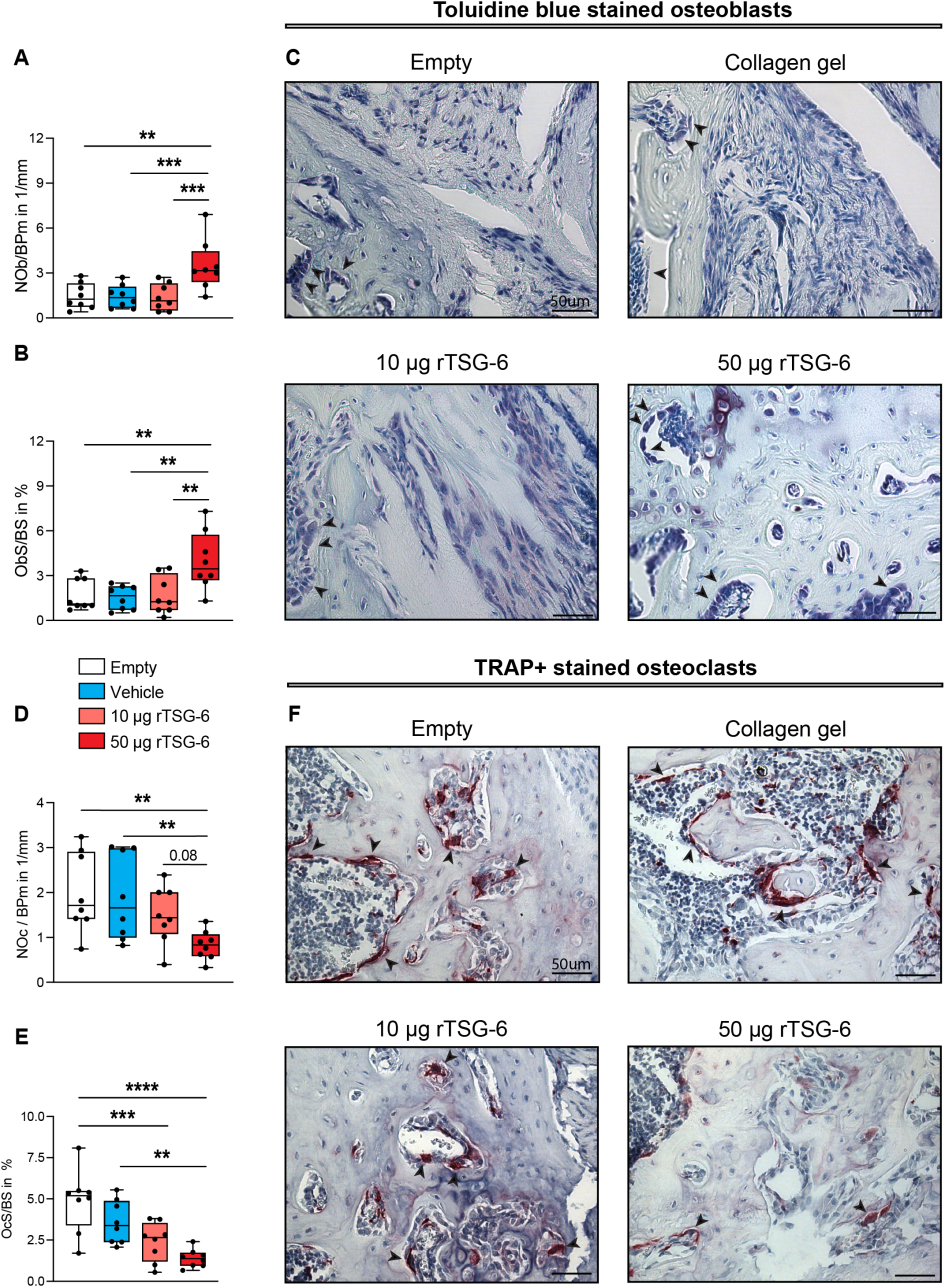
Histological analysis of fractured femurs on day 35 post-osteotomy. **(A)** Number of osteoblasts per bone perimeter (NOb/BPm), and **(B)** osteoblast surface per bone surface (ObS/BS), as determined by Toluidine blue staining within the defect area of fractured femurs. **(C)** Representative images of the fracture gap stained with Toluidine blue (arrowheads indicate osteoblasts). **(D)** Number of osteoclasts per bone perimeter (NOc/BPm), and **(E)** osteoclast surface per bone surface (OcS/BS), as determined by Tartrate-Resistant Acid Phosphatase (TRAP) staining within the defect area of fractured femurs. **(F)** Representative images from the fracture gap stained with TRAP (arrowheads indicate osteoclasts). n = 8 per group. Statistical differences between the groups were determined using ANOVA with Fisher’s LSD *post hoc* test. **p < .01, ***p < .001, ****p < .0001.

Next, we tested whether local delivery of rTSG-6 promotes bone repair in mice. rTSG-6 was embedded in a collagen gel and applied to femoral critical-sized defects (**Figure 5A**). Release kinetics showed that 93% of the rTSG-6 protein was released from the collagen gel within the first four days *in vitro* (**Supplementary Figure 4**). To determine the optimal therapeutic dose of rTSG-6 for fracture healing, we conducted a pilot study evaluating two different doses (10 and 50 µg) (**Figure 5A**). At day 35 post-surgery, the empty control and collagen-only groups developed typical atrophic non-unions with nearly closed cortical ends, while rTSG-6-treated groups exhibited new bone formation in the defect area, most prominent in the 50 μg rTSG-6-treated group (**Figures 5B-E**). Quantitative μCT analysis confirmed these findings, as the 50 μg rTSG-6 group exhibited a significantly higher bone volume within the defect area compared to those treated with 10 μg rTSG-6 and both control groups (**Figure 5F**). Supporting these findings, 75% of bone defects healed in the 50 μg rTSG-6 group, compared to 50% in the 10 μg rTSG-6 group, 37.5% in the collagen group, and 12.5% in the empty defect group (**Figures 5G-H**). Consistent with these findings, we found a significantly increased osteoblast number and surface in 50 μg rTSG-6-treated mice, compared to 10 μg rTSG-6-treated mice and both control groups (**Figures 6A-C**). Moreover, 50 μg rTSG-6-treated mice displayed a significantly reduced osteoclast number and surface compared to both control groups, with a trend toward reduction compared to the 10 µg rTSG-6 treated group (**Figures 6D-F**). In addition, osteoclast surface was significantly reduced in the 10 µg treated group compared to the empty control group (**Figure 6E**). In summary, these findings suggest that the high dose of 50 μg rTSG-6 significantly enhanced the healing of critical-sized bone defects, which is why subsequent experiments were conducted using the 50 μg rTSG-6 dose.

### 3.5 rTSG-6 treatment induces M2 macrophage polarization and T cell response in the fracture hematoma

**Figure 7:**
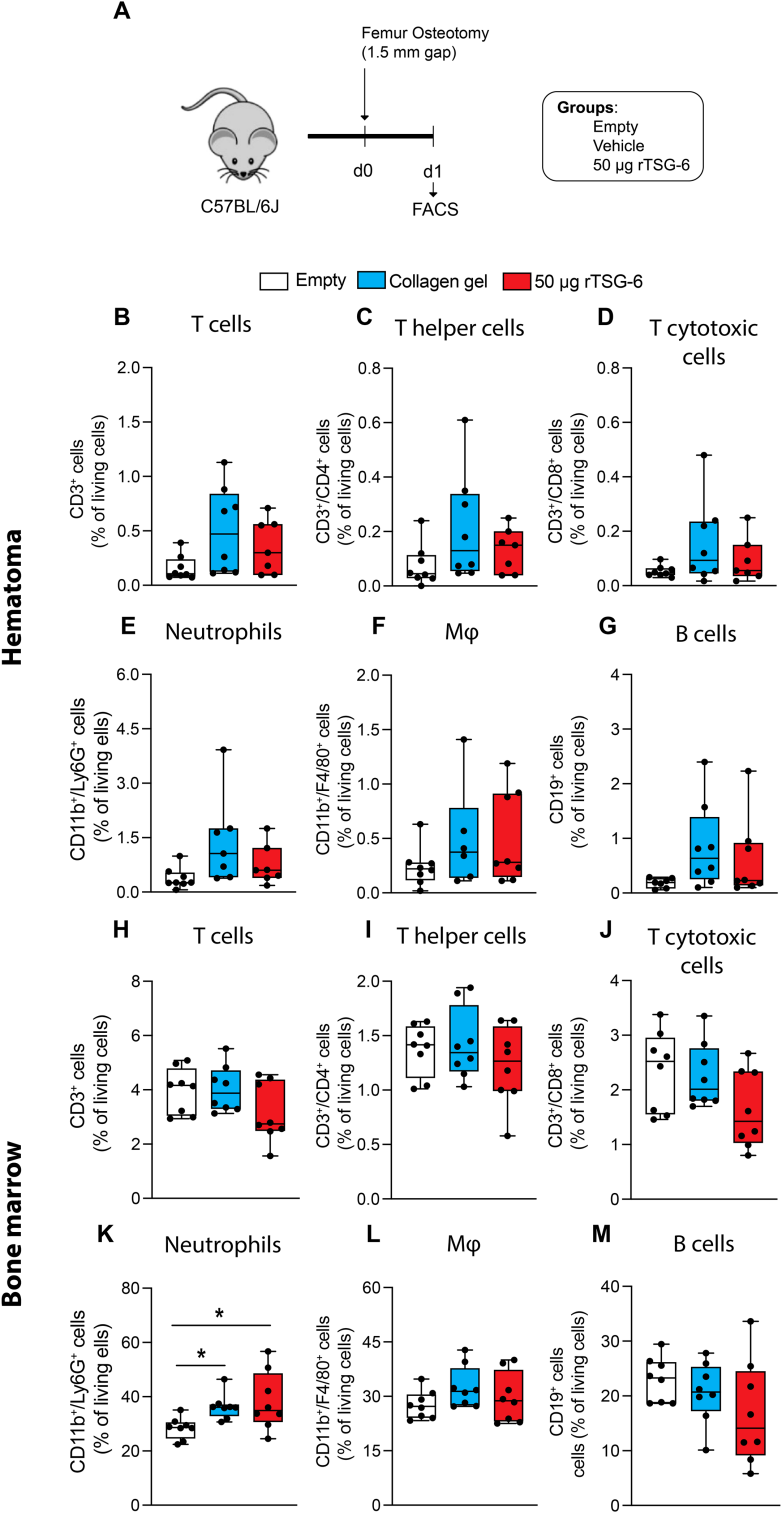
Flow cytometry analysis of innate and adaptive immune cell populations within the fracture hematoma and the contralateral bone marrow of mice on day 1 after fracture. **(A)** Experimental design. Percentage of living **(B, H)** T lymphocytes (CD3^+^), **(C, I)** T-helper lymphocytes (CD3^+^/CD4^+^), **(D, J)** cytotoxic T lymphocytes (CD3^+^/CD8^+^), **(E, K)** neutrophils (CD11b^+^/Ly6G^+^), **(F, L)** macrophages (CD11b^+^/F4/80^+^), and **(G, M)** B lymphocytes (CD19^+^) in the fracture hematoma and bone marrow. n = 8 per group. Statistical differences between the groups were determined using ANOVA with Fisher’s LSD *post hoc* test. *p < .05.

**Figure 8:**
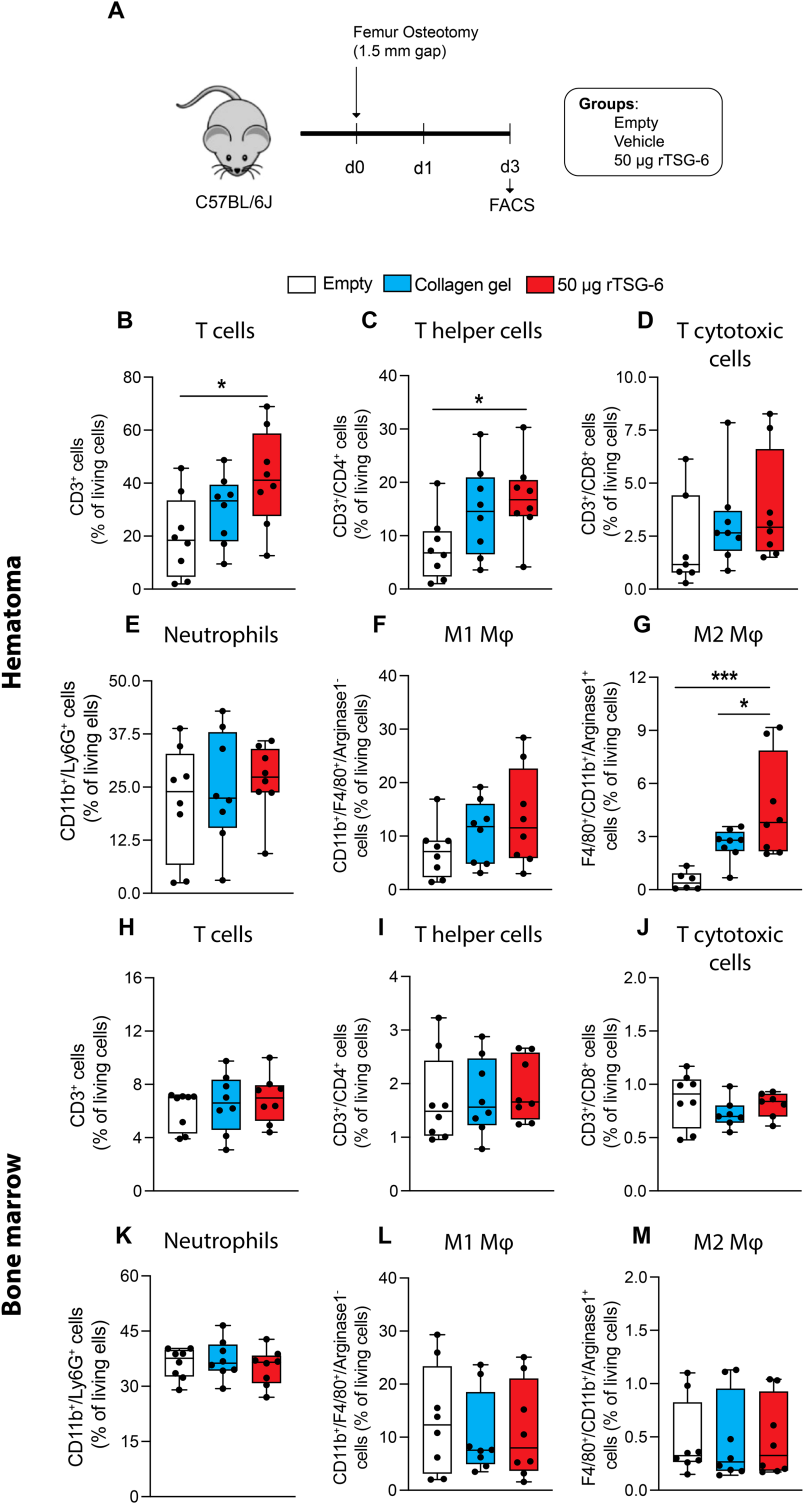
Flow cytometry analysis of innate and adaptive immune cell populations within the fracture hematoma and the contralateral bone marrow of mice on day 3 after fracture. **(A)** Experimental design. Percentage of living **(B, H)** T-lymphocytes (CD3^+^), **(C, I)** T-helper-lymphocytes (CD3^+^/CD4^+^), **(D, J)** cytotoxic T-lymphocytes (CD3^+^/CD8^+^), **(E, K)** neutrophils (CD11b^+^/Ly6G^+^), **(F, L)** pro-inflammatory M1 macrophages (CD11b^+^/F4/80^+^/Arginase1^-^), and **(G, M)** anti-inflammatory M2 macrophages (CD11b^+^/F4/80^+^/Arginase1^+^) in the fracture hematoma and bone marrow. n = 8 per group. Statistical differences between the groups were determined using ANOVA with Fisher’s LSD *post hoc* test. *p < .05, ***p < .001.

Finally, we investigated whether 50 μg rTSG-6 treatment affected innate and adaptive immune cell populations in the fracture hematoma, bone marrow and spleen during the early phase of fracture healing (**Figure 7A**). On day 1 post-surgery, no significant differences were observed in innate and adaptive immune cell populations within the fracture hematoma, bone marrow, or spleen across groups (**Figures 7B-M**, **Supplementary Figure 5**), except for significantly increased neutrophil numbers in the bone marrow of the 50 μg rTSG-6 and collagen-treated groups compared to the empty defect control (**Figure 7K**). On day 3 post-surgery, 50 μg rTSG-6 significantly increased the number of T-lymphocytes in the fracture hematoma compared to the empty defect group but not compared to the collagen gel control (**Figures 8A, B**). Within this population, the number of T-helper lymphocytes was significantly increased in 50 µg rTSG-6-treated mice compared to empty controls (**Figure 8C**). rTSG-6 also increased the number of anti-inflammatory M2 macrophages in the hematoma compared to both control groups, while other immune cell populations remained unchanged (**Figures 8D-G**). No changes were observed in the bone marrow or spleen at day 3 post-surgery (**Figures 8H-M**, **Supplementary Figure 6**). Furthermore, serum analysis revealed no significant changes in C-X-C motif chemokine ligand 5 (Cxcl-5), interferon-γ (IFN-γ), C-C-chemokine ligand 5 (Ccl5), Ccl11, Cxcl11, and Ccl7 between the groups on days 1 and 3 (**Table 1**), suggesting rTSG-6 acts locally without affecting systemic inflammation. In summary, these findings showed that rTSG-6 enhanced fracture healing by locally modulating immune responses, specifically by promoting M2 macrophage polarization and increasing T-helper cell infiltration in the fracture hematoma.

**Table 1:** Multiplex cytokine analysis of serum of fractured mice.

Serum	Day 1			Day 3		
	Empty	Collagen	50 µg rTSG-6	Empty	Collagen	50 µg rTSG-6
Cxcl5	108 ± 81	81 ± 87	71 ± 72	54 ± 77	119 ± 124	465 ± 842
Ifn-γ	2.5 ± 1.2	3.4 ± 2.3	3.1 ± 2.8	2.5 ± 1.3	2.6 ± 1.7	2.9 ± 1.3
Ccl5	53 ± 28	59 ± 15	64 ± 32	18 ± 26	27 ± 43	44 ± 37
Ccl11	120 ± 46	106 ± 31	106 ± 36	127± 52	221 ± 61	235 ± 110
Cxcl1	53 ± 47	33 ± 9	15 ± 24	20 ± 16	25 ± 44	19 ± 21
Ccl7	160 ± 67	146 ± 55	174 ± 63	81 ± 28	99.1 ± 39.0	109 ± 46

Cxcl5, C-X-C motif chemokine 5; Ifn-γ, Interferon-gamma; Ccl5, CC-Chemokin-Ligand-5; Ccl11, CC-Chemokin-Ligand-11; Cxcl11, C-X-C motif chemokine 11; Ccl7, CC-Chemokin-Ligand-7.

## 4 Discussion

In this study, we demonstrated that TSG-6 functions as a positive regulator of MSC osteogenic differentiation, likely through the regulation of key genes involved in osteogenesis and bone formation. Moreover, we identified that the local application of 50 μg rTSG-6 significantly enhanced bone regeneration in critical-sized femoral defects in mice, accompanied by increased osteoblast and reduced osteoclast parameters. Furthermore, rTSG-6 modulated the local immune response at the fracture site by promoting M2 macrophage polarization and T-helper cell activation. Taken together, these findings indicate that TSG-6 supports bone repair through both immunomodulatory and pro-regenerative mechanisms.

The therapeutic potential of TSG-6 has been demonstrated in several rodent models of inflammatory disease, including corneal injury (17), chronic constriction injury (35), wound healing (19, 20, 36), traumatic brain injury (16), osteoarthritis (18), and dry eye disease (37). However, its role in bone metabolism, particularly in fracture healing remains incompletely understood. Here, we found that *Tnfaip6* gene expression was markedly upregulated during early osteogenic differentiation in mMSCs, while *Tnfaip6* deficiency significantly impaired early and late osteogenesis, indicating a positive role of TSG-6 in this process. Consistent with these findings, primary mMSCs isolated from *Tnfaip6^-/-^* mice lost their ability to differentiate into adipogenic, osteogenic, and chondrogenic lineages, while exogenous rTSG-6 treatment failed to restore osteogenic and chondrogenic differentiation (27). Moreover, single-cell RNA-sequencing of healthy and osteoporotic bone samples revealed that *TNFAIP6*^+^ osteoblasts were strongly associated with cytokine regulation, osteoblast differentiation, and bone remodeling, while their downregulation correlated with disrupted bone homeostasis and more severe osteoporosis (38). In contrast, *Tnfaip6^-/-^* mice displayed a high bone mass phenotype, suggesting an inhibitory role of Tsg-6 in bone homeostasis (26). Whether this phenotype is a direct consequence of *Tnfaip6* deficiency or secondary to systemic effects remains unclear. Concluding, despite the conflicting results, TSG-6 plays an important role in the regulation of osteogenesis. Supporting this, we found that TSG-6 protein was locally expressed in the fracture callus throughout the entire course of fracture healing, with peaks during endochondral ossification. At this stage, it was predominantly expressed in hypertrophic chondrocytes and bone-forming osteoblasts; however, these observations require further quantitative validation.

To further investigate the molecular mechanisms underlying TSG-6-induced stimulation of osteogenesis observed in our study, we performed unbiased RNA-seq analysis of *Tnfaip6-*deficient mMSCs. The top five *Tnfaip6-*regulated genes within the highly-enriched “extracellular matrix organization” pathway were *Mmp13*, *Dcn*, *Mme*, *Ibsp*, and *Grem1*. All were downregulated except for *Grem1*, which was upregulated. Notably, the downregulated genes are well-established positive regulators of MSC osteogenic differentiation and play an essential role in bone development and repair. For example, MMP13, a matrix metalloproteinase critical for matrix remodeling, has been shown to enhance osteogenic differentiation of mMSC *in vitro* (39) and promote bone formation *in vivo* (40). Consistent with our findings, *TNFAIP6* knockdown significantly reduced *MMP13* expression in human chondrocytes (41), and their expression levels were positively correlated in both rat and human cartilage (42). DCN, an extracellular matrix proteoglycan in bone, regulates angiogenesis, osteogenic differentiation and bone formation (43–45), and was also reduced following *TNFAIP6* deletion in human keratinocytes (46). MME, a zinc-dependent metallopeptidase, enhanced osteogenesis in human MSCs (47, 48) and its higher expression in bone of newborn compared with adult mice suggests a role in bone growth (49). IBSP, encoding bone sialoprotein, serves as an early mineralization marker during osteogenic differentiation (50). Impaired bone repair has been reported in BSP-deficient mice (51, 52) whereas recombinant BSP improved bone healing in rats (53, 54). Conversely, *Grem1*, a BMP antagonist, was upregulated in *Tnfaip6-*deficient mMSCs, in line with its known inhibitory role in osteoblastogenesis and association with osteopenia (55–57). Together, these findings indicate that TSG-6 is a positive regulator of MSC osteoblast differentiation, acting through critical gene networks involved in bone formation. However, a limitation of our study is that these results were not validated *in vivo*, particularly through histological analysis, which should be addressed in future studies.

Based on our *in vitro* results and the known therapeutic potential of TSG-6, we sought to determine whether it could enhance bone fracture healing. Previous studies employed TSG-6 doses ranging from 1 to 50 µg delivered via intra-articular, intradermal, intraperitoneal, and subcutaneous injections (16, 18–20, 36). Based on our previous findings, demonstrating that 10 and 50 ng/mL TSG-6 suppressed TNF-α secretion from macrophages *in vitro* (19), and supported by extensive literature research, showing that 50 µg TSG-6 exerts immunomodulatory and pro-regenerative effects in rodent models of osteoarthritis (18), traumatic brain injury (16), and severe burn wounds (36), we conducted a pilot study to define the optimal dose for fracture healing. For this purpose, 10 µg and 50 µg rTSG-6 were locally delivered in a collagen-type 1 gel into the fracture gap. Treatment with 50 µg rTSG-6 significantly enhanced the healing of critical-sized femoral defects after 35 days, whereas 10 μg rTSG-6 was insufficient, thus being consistent with earlier reports (16, 18, 36). However, since only two concentrations were evaluated, we cannot determine whether the observed effect represents a “all-or-none” phenomenon or is dose-dependent, which constitutes a limitation of our study. Release studies demonstrated that rTSG-6 was largely liberated from the collagen gel within 48 h, suggesting that its effects likely occur during the early inflammatory phase. Indeed, by day 3 post-fracture, we observed increased numbers of anti-inflammatory M2 macrophages, T-lymphocytes, and T-helper lymphocytes in the hematoma, potentially explaining the improved healing outcome after 35 days. Macrophages are crucial regulators of bone healing. Pro-inflammatory M1 macrophages dominate the early inflammatory phase, by initiating inflammation and removing tissue debris, while their subsequent switch to anti-inflammatory M2 macrophages is essential for resolution of inflammation, angiogenesis, MSC recruitment and osteogenesis (58, 59). Impaired bone healing has been reported in aged rats with reduced M2 macrophages in the early fracture callus (60). The anti-inflammatory effects of TSG-6 described in several preclinical studies were often linked to an enhanced M2 macrophage polarization (23, 61). Mechanistically, TSG-6 interacts with macrophages via CD44 and reduces inflammation in mice by blocking NF-κB activation in macrophages (15). Therefore, the pro-regenerative effects of TSG-6 on bone healing may be mediated, at least in part, through macrophage-depended mechanisms. However, as the underlying mechanism remains unclear, functional assays, such as gain- or loss-of-function experiments and the use of NF-κB pathway inhibitors, would provide valuable mechanistic insights into how TSG-6 regulates M2 macrophage polarization, particularly in the context of bone repair. This warrants further investigation. In addition to macrophages, T-lymphocytes play critical roles in fracture healing (62). For example, regulatory T cells (Tregs) support osteogenesis by suppressing excessive inflammation and promoting MSC differentiation toward the osteogenic lineage (63). Similarly, γδ T cells support the recruitment of osteoprogenitor cells and fracture callus formation (64). T-helper 2 (Th2) cells contribute to bone repair by promoting M2 macrophage polarization, angiogenesis and matrix remodeling (65). In a murine colitis model, MSC-derived TSG-6 reduced the differentiation of T-helper cells and increased Tregs (66). Thus, the beneficial effects of TSG-6 on bone healing may also involve T-helper cell-mediated pathways, warranting further investigation into the underlying mechanisms. However, our analysis was limited to T-helper and cytotoxic T cells, while other T cell subsets were not assessed. Moreover, T-helper cell numbers did not differ significantly compared with the collagen gel control, which may reflect intrinsic T cell regulatory properties of collagen itself (67). Additional cytokine and chemokine profiling of the fracture hematoma, particularly focusing on the local assessment of cytokine signatures associated with M1/M2 macrophage (e.g., IL-4, IL-10, IL-13, TGF-β) and T helper cell polarization (IL-12, IL-1β, IL-4, IFN-γ), at either gene or protein level, will be necessary to further characterize the immunomodulatory mechanisms of TSG-6 *in vivo*. We did not detect differences in B cell and neutrophil numbers in the fracture hematoma following TSG-6 treatment. Interestingly, neutrophil numbers were increased in the bone marrow of rTSG-6-treated mice. These findings contrast with previous reports that TSG-6 inhibits neutrophil migration by binding with high affinity to CXCL8 and CCL2 (22, 24). However, as no systemic differences in serum cytokine levels were observed, the increased neutrophils in the bone marrow likely do not reflect systemic effects of locally applied rTSG-6. In summary, local delivery of rTSG-6 modulated the early immune response in the fracture hematoma, especially M2 macrophage polarization and T-helper cell response, thereby likely contributing to improved bone healing. However, a limitation of our study is that we examined only male mice, even though both immune responses and bone healing are known to be influenced by sex (68). Fracture healing is generally faster in male mice than in female mice (69). Estrogen-deficiency exacerbates fracture-induced inflammation and impairs fracture healing (70, 71), whereas male mice experience greater posttraumatic bone loss associated with higher TNF-α levels (72). Interestingly, MSCs isolated from females express higher baseline levels of TSG-6 compared to those isolated from males (73), while the protective effects of TSG-6 on hypoxic-ischemic brain injury are more pronounced in male mice (74). Taken together, these observations suggest that future studies should investigate whether TSG-6 exerts similar protective effects in female mice.

Fracture calli of mice treated with 50 µg rTSG-6 contained significantly more newly formed bone, accompanied by increased osteoblast and reduced osteoclast parameters compared to controls on day 35 post-surgery. Our *in vitro* findings, along with previous studies, indicate that TSG-6 acts as a positive regulator of osteogenesis and bone formation (26, 27), consistent with the enhanced bone healing observed *in vivo*. Regarding osteoclasts, it has been shown that osteoclast precursors isolated from the bone marrow of *Tnfaip6^-/-^* mice displayed enhanced resorptive activity compared to wild-type controls (26). In line, rTSG-6 inhibits osteoclastogenesis of human osteoclast precursors in a dose-dependent manner (75). In agreement with these findings, *Tnfaip6^-/-^* mice developed more severe cartilage proteoglycan-induced arthritis, marked by excessive cartilage degradation and bone erosion, indicating that TSG-6 suppresses osteoclast-mediated bone resorption under inflammatory conditions (76). Together, these findings suggest that TSG-6 regulates osteoclastogenesis by exerting inhibitory effects, particularly under inflammatory conditions.

In conclusion, our study demonstrated that local administration of 50 µg rTSG-6 enhanced bone regeneration in a murine critical-sized femoral defect model by promoting osteoblastogenesis, reducing osteoclast activity, and modulating the immune response, most notably by inducing M2 macrophage polarization and influencing T-helper cell response. *Tnfaip6* knockdown *in vitro* impaired the osteogenic differentiation of mMSCs, which was associated with changes in the expression of key genes involved in osteogenesis and bone formation, underscoring the role of TSG-6 as a positive regulator of osteoblastogenesis. Together, these findings identify TSG-6 as a promising candidate for immunomodulatory and regenerative therapies across a broad range of bone pathologies, particularly in conditions where bone formation requires support. This includes metabolic bone diseases such as osteoporosis, as well as traumatic and degenerative conditions including fractures. Herein, TSG-6 might be especially relevant in patients who are known to be at high risk for fracture healing complications, such as the elderly and those with osteoporosis, concomitant trauma or large bone defects. Future mechanistic studies are warranted to delineate the molecular pathways underlying TSG-6 activity during fracture healing, to elucidate its role in other bone pathologies, and to evaluate its translational potential in clinical settings.

## Data Availability

The original contributions presented in the study are publicly available. This data can be found here: https://doi.org/10.5281/zenodo.17176977.

## Supplementary material

SUPPLEMENTARY DATA SHEET 1 Supplementary Results.

Supplementary Table 1 Read Counts siNT vs siTSG6 Whole Data.

Supplementary Table 2 DEGs siNT vs siTSG6 Whole Data.

Supplementary Table 3 Metascape Pathways Analysis.
