# Supplementary material for: TSG-6 promotes healing of critical-sized bone defects in mice
Source: Front Immunol. 2025 Nov 28;16:1712152. doi: 10.3389/fimmu.2025.1712152 (PMC12700086; doi:10.3389/fimmu.2025.1712152)
Supplement: SUPPLEMENTARY DATA SHEET 1 — Supplementary Results. [file DataSheet1.pdf]

Supplementary Material

1 Supplementary Figures

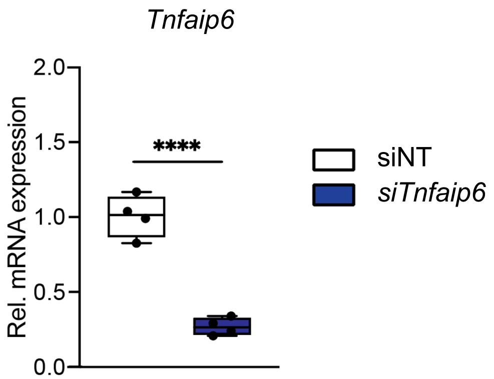

Supplementary Figure 1. *Tsg-6* (*Tnfaip6*) knockdown efficiency after 48 h of reverse transfection. Tumor necrosis factor-stimulated gene 6 *(Tnfaip6)* was knocked down by incubating murine mesenchymal stem cells (mMSCs) with siRNA targeting *Tnfaip6* (*Tsg-6*) for 48 h. Non-targeting siRNA (siNT) served as negative control. n = 4 per group. Statistical differences between two groups were determined using unpaired Student's *t*-test. ****$p < .0001$.

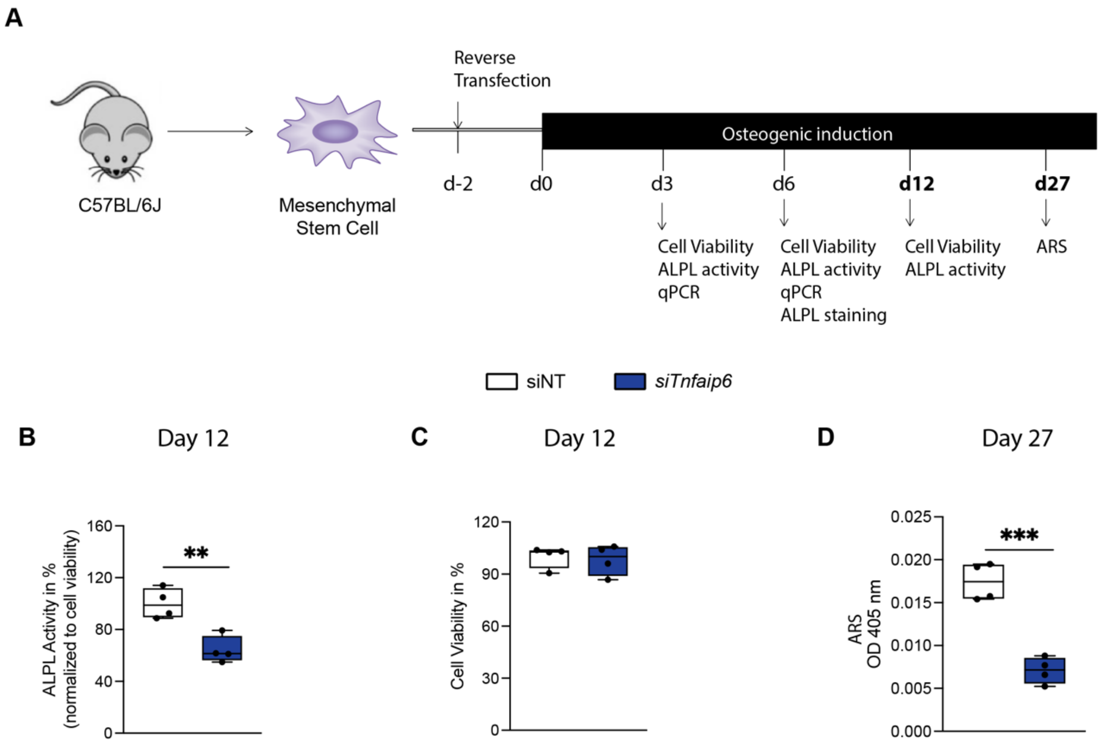

Supplementary Figure 2. Effect of siRNA-mediated *Tsg-6* (*Tnfaip6*) knockdown on late osteogenic differentiation of mMSCs. (A) Experimental design. (B) Quantitative alkaline phosphatase (ALP) staining, and (C) relative cell viability on day 12 of osteoblast differentiation. (D) Quantitative Alizarin Red S (ARS) staining on day 27 of osteoblast differentiation. n = 4 per group. Statistical differences between two groups were determined using unpaired Student's *t*-test. $^{**}p < .01$, $^{***}p < .001$.

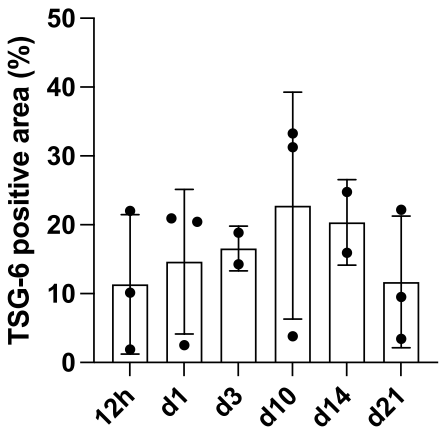

Supplementary Figure 3. Positive TSG-6 staining (%) locally at the fracture site during the time course of fracture healing analyzed using Fiji software. N = 2-3 per time points.

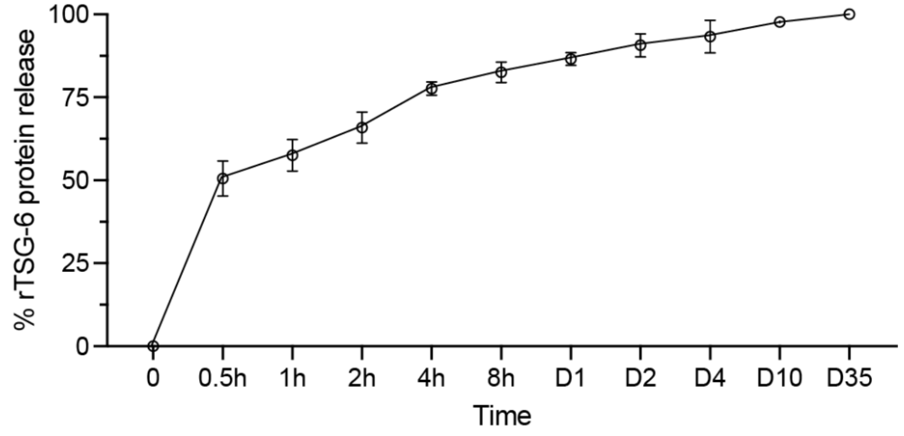

Supplementary Figure 4. Recombinant tumor necrosis factor-stimulated gene-6 (rTSG-6) release kinetic. 1 µL rTSG-6 [1 mg/mL] was loaded into a collagen gel and incubated in phosphate buffered saline at 37°C under constant agitation. The supernatant was collected at defined time points (30 min, 1 h, 2 h, 4 h, 8 h, 1 d, 2 d, 4 d, 10 d, 35 d). n = 3 per group.

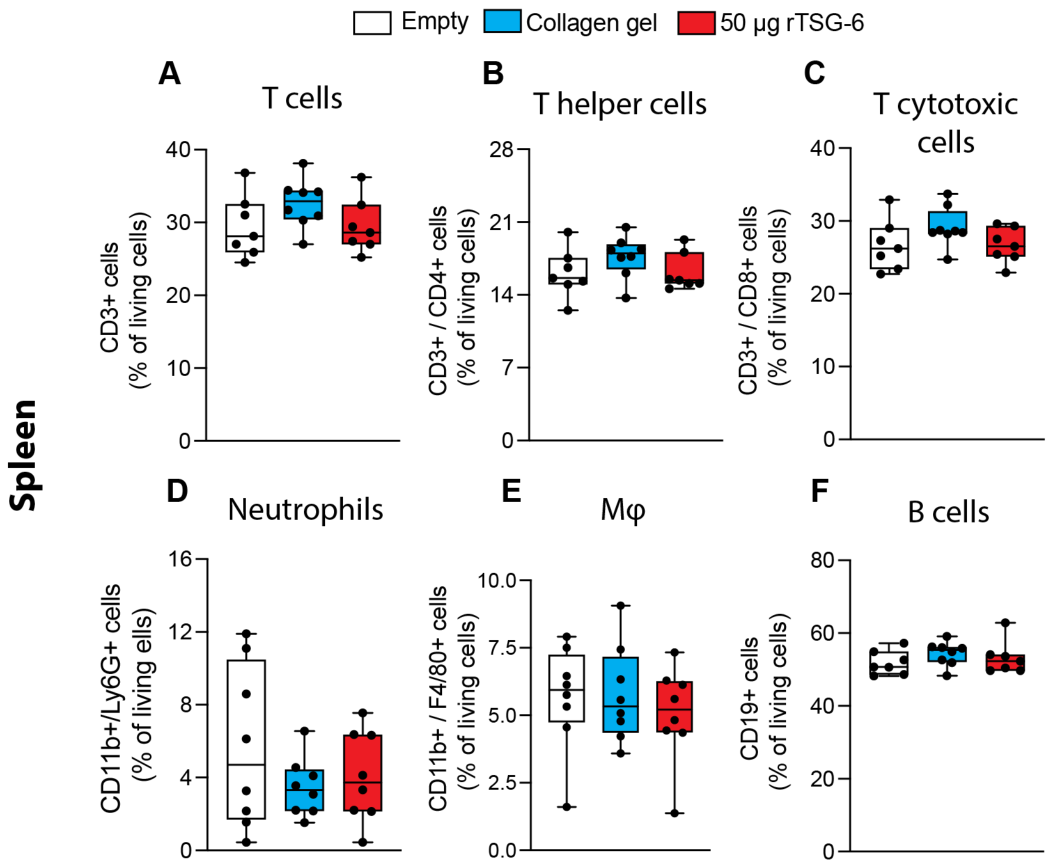

Supplementary Figure 5. Flow cytometry analysis of innate and adaptive immune cell populations within the spleen of mice on day 1 after fracture. Percentage of living (A) T lymphocytes ($CD3^+$), (B) T-helper lymphocytes ($CD3^+/CD4^+$), (C) cytotoxic T lymphocytes ($CD3^+/CD8^+$), (D) neutrophils ($CD11b^+/Ly6G^+$), (E) macrophages ($CD11b^+/F4/80^+$), and (F) B lymphocytes ($CD19^+$) in the spleen. n = 8 per group.

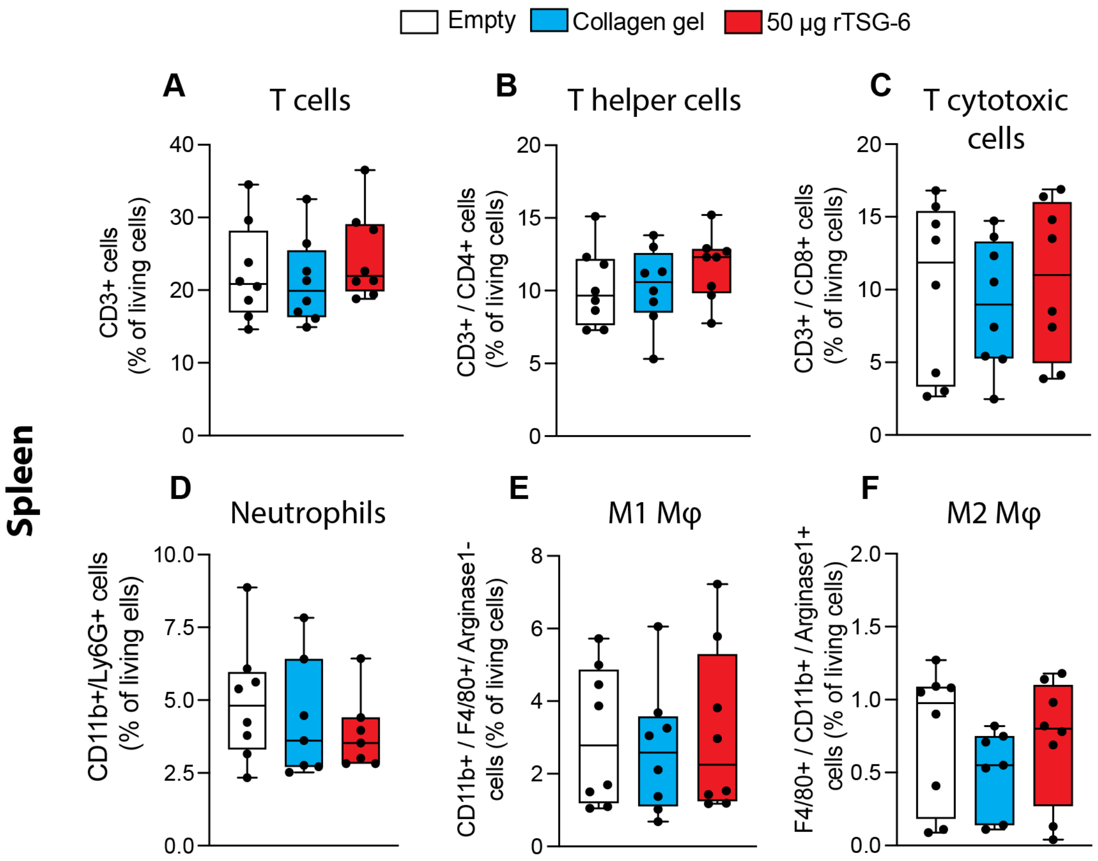

Supplementary Figure 6. Flow cytometry analysis of innate and adaptive immune cell populations within the spleen of mice on day 3 after fracture. Percentage of living (A) T lymphocytes ($CD3^+$), (B) T-helper lymphocytes ($CD3^+/CD4^+$), (C) cytotoxic T lymphocytes ($CD3^+/CD8^+$), (D) neutrophils ($CD11b^+/Ly6G^+$), (E) M1 macrophages ($CD11b^+/F4/80^+/Arginase1^-$), and (F) M2 macrophages ($CD11b^+/F4/80^+/Arginase1^+$) in the spleen. n = 8 per group.

2 Supplementary Tables

Supplementary Table 1. Murine primer sequences for qPCR analysis.

Gene symbol	Forward primer (5´-3´)	Reverse primer (5´-3´)
Actb	TGAAGTGTGACGTTGACATCC	CTTCTGCATCCTGTCAGCAA
Tnfaip6	CCCACATGCAAAGGAGTGTG	TGAGCCGAATGTGCCAGTAG
Alpl	GCTGATCATTCCCACGTTTT	GAGCCAGACCAAAGATGGAG
Runx2	CCACCACTCACTACCACACG	CACTCTGGCTTTGGGAAGAG
Sp7	CCTTAACCCAGCTCCCTACC	ACCGCCTTGGGCTTATAGAC
Bglap	GCGCTCTGTCTCTCTGACCT	ACCTTATTGCCCTCCTGCTT

Beta-actin (*Actb*), tumor necrosis factor-stimulated gene 6 (*Tnfaip-6*), alkaline phosphatase (*Alpl*), runt-related transcription factor 2 (*Runx2*), SP7 transcription factor (*Sp7*), bone gamma-carboxyglutamate protein (*Bglap*).

Supplementary Table 2. Collagen gel preparation.

Compounds	Vehicle	10 µg rTSG-6	50 µg rTSG-6
**Gel neutralization solution (5x)**	10 µL	10 µL	10 µL
**PBS**	15 µL	5 µL	5 µL
**rTSG-6**	-	10 µL [1 mg/mL]	10 µL [5 mg/mL]
**Collagen [10 mg/ml]**	25 µL	25 µL	25 µL
**Final volume**	**50 µL**	**50 µL**	**50 µL**

Phosphate buffered saline (PBS), recombinant tumor necrosis factor-stimulated gene 6 (rTSG-6).

Supplementary Table 3. Antibodies used for fluorescent-activated cell sorting (FACS).

Antibody	Fluorochrome	Dilution	Company	Product
**F4/80 Rat anti-mouse**	FITC	1:50	Affymetrix eBioscience	11-4801-82
**Rat IgG2a K isotype control**	FITC	1:50	Affymetrix eBioscience	11-4321-42
**CD3e American hamster anti-mouse**	PE-Cyanine7	1:100	Affymetrix eBioscience	25-0031-82
**American hamster IgG isotype control**	PE-Cyanine7	1:100	Affymetrix eBioscience	145-2C11
**CD8a Rat anti-mouse**	APC	1:800	Affymetrix eBioscience	17-0081-81
**Rat IgG2a K isotype control**	APC	1:800	Affymetrix eBioscience	17-4321-81
**CD4 Rat anti-mouse**	APC-eFluor® 780	1:200	Affymetrix eBioscience	47-0041-82
**Rat IgG2b K isotype control**	APC-eFluor® 780	1:200	Affymetrix eBioscience	47-4031-82
**CD11b Rat anti-mouse**	Alexa Fluor® 700	1:400	Affymetrix eBioscience	56-0112-80
**Rat IgG2a K isotype control**	Alexa Fluor® 700	1:400	Affymetrix eBioscience	56-4031-80
**CD19 Rat anti-mouse**	PE	1:400	BioLegend	152409
**Rat IgG2a K isotype control**	PE	1:400	BioLegend	400607
**Ly-6G Rat anti-mouse**	V450	1:400	BD Pharmingen	560603
**Rat IgG2a K isotype control**	V450	1:400	BD Pharmingen	560377
**Arginase 1 Rabbit anti-mouse**	-	1:200	Bioss	bs-8585R
**Goat anti-rabbit**	PE	1:500	Abcam	bs-72465
**7-Aminoactinomycin**	7-AAD	1:100	Sigma	A 9400

Fluorescein (FITC), phycoerythrin (PE), allophycocyanin (APC), 7-Aminoactinomycin (7AAD).
